# Effect of 6-gingerol on proliferation and apoptosis of ovarian cancer cells by the PI3K/AKT/mTOR pathway

**DOI:** 10.1016/j.bbrep.2026.102717

**Published:** 2026-07-18

**Authors:** Ai-Ying Guo, Yong-Shuai Chen, Huan Li, Yun-Yun Ren, Xiao-Qing Tan, Xi-Yu Zhang, Jun Xiong

**Affiliations:** a Department of Obstetrics and Gynecology, The Second Affiliated Hospital of Nanchang University, Nanchang, 330006, China; b Fuzhou Medical College of Nanchang University, Fuzhou, 344000, China; c The Second Clinical Medical College of Nanchang University, Nanchang, 330006, China; d Queen Mary College of Nanchang University, Nanchang, 330006, China; e Nanchang University, Nanchang, 330006, China; f Affiliated Rehabilitation Hospital, Jiangxi Medical College, Nanchang University, Nanchang, 330006, China

**Keywords:** 6-Gingerol, Ovarian cancer, PI3K/AKT/mTOR pathway, Apoptosis

## Abstract

**Background:**

As a major contributor to mortality among gynecological malignancies, ovarian cancer highlights an urgent need for novel therapeutic approaches. Although 6-gingerol—a primary bioactive compound found in ginger—is recognized for its anticancer properties, its precise role and underlying mechanisms in ovarian cancer remain largely unexplored.

**Methods:**

SKOV3 ovarian cancer cells were treated with varying concentrations of 6-gingerol, either alone or in combination with Recilisib, an activator of the PI3K signaling pathway. Cell viability was evaluated using the MTT assay, whereas apoptosis and cell cycle distribution were assessed via flow cytometry. Western blot analysis was conducted to measure protein levels of cyclin E, cyclin B, Beclin1, Atg7, and P62.

**Results:**

6-gingerol treatment led to a concentration-dependent reduction in cell viability, induced G2-M phase arrest, and promoted apoptosis. These changes were associated with elevated expression of Beclin1 and Atg7, alongside decreased levels of cyclin E, cyclin B, and P62 (p < 0.05). Notably, co-treatment with Recilisib significantly counteracted the effects of 6-gingerol on cell viability, cell cycle regulation, apoptosis, and the expression of autophagy-related proteins (p < 0.05).

**Conclusion:**

These findings indicate that 6-gingerol may exert antitumor activity in ovarian cancer cells through inhibition of the PI3K/AKT/mTOR pathway, resulting inreduced cell viability, enhanced apoptosis, and increased autophagic activity. The pivotal involvement of this signaling cascade is supported by the observation that its targeted activation effectively reverses the actions of 6-gingerol. Collectively, these results support further investigation into the biological effects of 6-gingerol in ovarian cancer models.

## Highlights

• First evidence linking 6-Gingerol to ovarian cancer autophagy by PI3K/AKT/mTOR.
• Induces G2-M phase arrest and apoptosis in SKOV3 cells dose-dependently.
• Autophagy is enhanced by 6-Gingerol via upregulation of Beclin1 and Atg7, and downregulation of P62.
• **PI3K activator Recilisib reverses 6-Gingerol's anti-tumor effects.**
• **These findings support 6-Gingerol as a promising natural compound for targeted therapy in ovarian cancer**.

## 1 Introduction

Ovarian cancer is one of the most common malignant tumors affecting women worldwide. Its insidious onset and difficulty in early diagnosis often lead to a generally poor prognosis. In recent years, although some high-income countries have witnessed a declining trend in ovarian cancer incidence, the disease burden in China remains severe, with mortality rates continuing to rise [1]. Therefore, exploring novel therapeutic strategies to improve the survival outcomes of patients with ovarian cancer holds significant clinical importance. [2]. Ginger contains a variety of bioactive phenolic substances, such as gingerol, paradol, shogaol, singol, etc. Among them, the content of 6-gingerol is the highest. [3]. 6-Gingerol, a principal bioactive component of ginger, possesses antioxidant, antiplatelet, anti-inflammatory, and antiproliferative activities [4]. Earlier research has indicated that 6-gingerol suppresses mTOR activity by inhibiting PI3K/AKT phosphorylation, ultimately leading to cell cycle arrest and apoptosis in cancer cells [5]. As a member of the intracellular phosphatidylinositol kinase family, PI3K serves a crucial function in relaying signals from the external environment to the interior of the cell. Dysregulated activation of this signaling axis ranks among the most frequent molecular alterations observed across various human malignancies. [6]. However, it remains unclear whether 6-gingerol exerts its effects through direct interaction with PI3K signaling. In this study, in vitro experiments were conducted to evaluate the impact of this compound on ovarian cancer cell proliferation, apoptotic processes, and cell cycle dynamics, along with an exploration of the associated molecular mechanisms. Our findings revealed a robust link between PI3K pathway activity and 6-gingerol-induced apoptosis. Accordingly, further investigation was undertaken to elucidate how this bioactive component modulates proliferation, apoptosis, and autophagic activity in ovarian cancer cells via the PI3K/AKT/mTOR axis, aiming to further explore the potential molecular mechanisms underlying its biological effects in ovarian cancer cells [7].

## 2 Materials and methods

### Ethical statement

This study utilized the human ovarian cancer cell line SKOV3, which was obtained from the American Type Culture Collection (ATCC). All experiments were conducted in vitro and did not involve human participants, animal subjects, or clinical samples. Therefore, ethical approval from an institutional review board and informed consent were not required. The research adhered strictly to international standards for the use of cell lines in scientific investigations.

### 2.1 Cells and major reagents

The human ovarian adenocarcinoma cell line SK-OV-3 was obtained from the American Type Culture Collection (ATCC, Manassas, VA, USA; catalogue number: HTB-77™). The SK-OV-3 cells were authenticated by short tandem repeat (STR) profiling within 3 years before use in the present experiments, and the STR profile was confirmed to match the reference profile of SK-OV-3 cells. The cells were also tested for mycoplasma contamination before the experiments using a mycoplasma detection assay, and only mycoplasma-negative cultures were used. Cells were maintained in Dulbecco's Modified Eagle Medium (DMEM) supplemented with 10% fetal bovine serum under standard culture conditions.Dulbecco's Modified Eagle Medium (DMEM) was obtained from Life Technologies (Grand Island, NY, USA). Fetal bovine serum (FBS) was purchased from PAN Biotech (GmbH, Aidenbach, Germany). Antibodies against cyclin E, cyclin B, Beclin1, Atg7, P62, beta-actin, and caspases were acquired from Cell Signaling Technology (Beverly, MA, USA), while the antibody against poly ADP ribose polymerase (PARP) was sourced from Santa Cruz Biotechnology (Santa Cruz, CA). 6-Gingerol was obtained from MedChemExpress (Shanghai, China). The primary antibodies used for Western blotting included antibodies against cyclin E, cyclin B, Beclin1, Atg7, P62, β-actin, Bax, cleaved-PARP, PI3K, p-PI3K, AKT, p-AKT, mTOR, and p-mTOR. Detailed information regarding the antibody manufacturer, host species, and working dilution is provided in Table S1.

### 2.2 Cell culture

SKOV3 cells were cultured in DMEM supplemented with 10% fetal bovine serum in a humidified incubator at 37°C with a 5% CO_2_ atmosphere. The medium was replaced every two days, and cells in the logarithmic phase of growth were used for subsequent experiments.

### 2.3 Detection of cell viability by MTT assay

SKOV3 cells were seeded into 96-well plates at a density of 2 × 10^4^ cells/well in 200 μL of medium. After 24 h, cells were treated with 6-Gingerol at concentrations of 0, 2.5, 5.0, 10.0, 20.0, 40.0, 80.0, 160.0, and 320.0 mg/L. A control group was included, with three replicate wells for each concentration. After 48 h of incubation, cell viability was measured using the MTT assay. The absorbance (A) at 570 nm was recorded, and the cell viability inhibition rate was calculated using the formula:

Cell viability Inhibition Rate (%) = 1 - (A_experimental / A_control) × 100%.

### 2.4 Detection of cell cycle distribution and apoptosis by flow cytometry

For cell cycle analysis, SKOV3 cells were seeded into 6-well plates at 1 × 10^6^ cells/well and treated with 6-Gingerol at concentrations of 0, 5.0, 20.0, and 40.0 mg/L for 48 h. After harvesting, cells were washed twice with PBS, fixed in pre-cooled 75% ethanol for 6 h at 4°C, and then stained with propidium iodide (PI) and RNase A. Cell cycle distribution was assessed using flow cytometry.

For apoptosis analysis, SKOV3 cells underwent a 48-h exposure to 6-Gingerol, after which they were washed with PBS and re-suspended in binding buffer at a density of 1 × 10^6^ cells/mL. The resulting cell suspension was then mixed with 5 μL of Annexin V-FITC (Santa Cruz Biotechnology) and incubated for 10 min. Subsequently, 5 μL of propidium iodide (PI) was added, and the mixture was kept in the dark for an additional 5 min. Following this, 500 μL of PBS was introduced, and apoptotic rates were determined via flow cytometric analysis.

### 2.5 Western blot analysis was performed to assess protein levels of cyclin E, cyclin B, Beclin1, Atg7, P62, and components of the PI3K signaling pathway in treated cells

SKOV3 cells were treated with 6-Gingerol for 48 h, after which the cells were lysed with cell lysis buffer on ice for 30 min. The supernatant was collected by centrifugation, and protein concentration was determined using the bicinchoninic acid (BCA) method. Equal amounts of protein were mixed with loading buffer, denatured, and separated by SDS-PAGE. Proteins were transferred to a nitrocellulose membrane and blocked with 5% skim milk. Detailed information on the primary and secondary antibodies used for Western blotting, including antibody source, host species, and working dilution, is provided in Supplementary Table S1. After washing, the membranes were incubated with the corresponding primary antibodies at the indicated dilutions overnight at 4°C, followed by incubation with HRP-conjugated secondary antibodies at room temperature for 1 h. β-actin was used as the internal loading control for total protein expression, whereas phosphorylated PI3K, AKT, and mTOR were normalized to their corresponding total protein levels.Chemiluminescent detection was used to visualize protein bands, and band intensity was analyzed using a gel imaging system. Protein expression levels were quantified relative to β-actin.

### 2.6 Effect of PI3K/AKT/mTOR pathway activator on proliferation, apoptosis, and cell cycle distribution in 6-gingerol-Treated SKOV3 cells

SKOV3 cells were cultured in 6-well plates for 48 h and treated with 20.0 mg/L 6-Gingerol. Subsequently, the cells were exposed to a PI3K/AKT/mTOR pathway activator for 2 h. Proliferation, apoptosis, and cell cycle distribution were assessed, along with the expression patterns of relevant proteins.

### 2.7 Statistical analysis

Data were analyzed using SPSS for Windows, version 17.0 (SPSS Inc., Chicago, IL, USA). All experiments were performed with three independent biological replicates, and each experiment included three technical replicates where applicable. Data are presented as mean ± standard deviation (SD). Before parametric statistical testing, the normality of data distribution and homogeneity of variance were assessed using the Shapiro-Wilk test and Levene's test, respectively. For comparisons between two independent groups, an unpaired two-tailed Student's t-test was used when the assumptions of normality and equal variance were satisfied. For comparisons among multiple groups, one-way analysis of variance (ANOVA) was performed. When a significant overall difference was detected by ANOVA, pairwise comparisons were conducted using the Student-Newman-Keuls post-hoc test. A two-sided p value < 0.05 was considered statistically significant.

## 3 Results

### 3.1 Inhibitory effect of 6-gingerol at different concentrations on the viability of ovarian cancer cells

As shown in Table 1, the full concentration-response data from the MTT assay are presented for SKOV3 cells treated with 6-gingerol at concentrations ranging from 0 to 320.0 mg/L for 48 h. 6-Gingerol inhibited SKOV3 cell viability in a concentration-dependent manner, with progressively increased inhibition rates observed at higher concentrations.The calculated IC_50_ value was 40.22 mg/L.

**Table 1:** Inhibitory effect of 6-Gingerol on the viability of ovarian cancer cells (x ± s, n = 9).

Groups	Cell inhibition rate (%)
6-Gingerol 0 mg/L	2.41 ± 0.32
6-Gingerol 2.5 mg/L	6.56 ± 0.98*
6-Gingerol 5.0 mg/L	15.05 ± 2.38*
6-Gingerol 10.0 mg/L	24.76 ± 3.93*
6-Gingerol 20.0 mg/L	36.13 ± 4.32*
6-Gingerol 40.0 mg/L	48.17 ± 4.24*
6-Gingerol 80.0 mg/L	61.88 ± 5.64*
6-Gingerol 160.0 mg/L	75.73 ± 5.04*
6-Gingerol 320.0 mg/L	89.77 ± 5.43*
F	545.139
P	0.000

Note: Compare with 6-Gingerol 0 mg/L group.

*p < 0.05.(p, probability value).

### 3.2 Effect of 6-gingerol on the cell cycle distribution of ovarian cancer cells

**Fig. 1:**
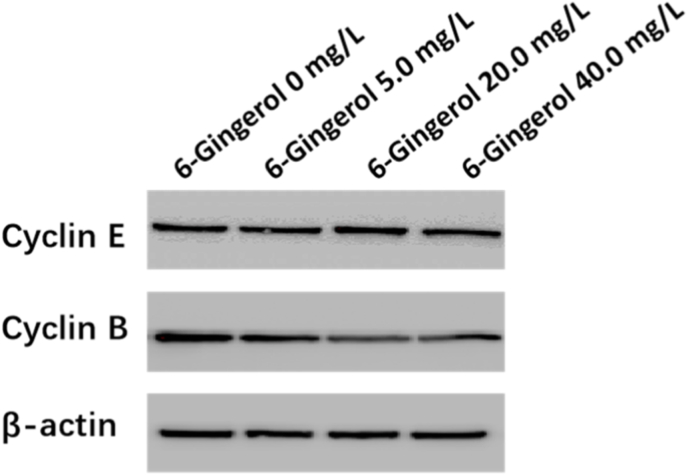
Representative Western blot images showing cyclin E and cyclin B protein expression in SKOV3 ovarian cancer cells after treatment with 6-gingerol at 0, 5.0, 20.0, and 40.0 mg/L for 48 h. β-actin was used as the internal loading control. The corresponding normalized quantitative data are presented in Table 2.

As shown in Table 2 and Fig. 1, exposure to 6-Gingerol led to a marked elevation in the proportion of SKOV3 cells arrested at the G2-M phase, accompanied by a significant reduction in the percentages of cells residing in both G1 and S phases (p < 0.05*). Protein levels of cyclin E and cyclin B were also substantially diminished following treatment (*p < 0.05). Moreover, these alterations in cell cycle distribution and the accompanying suppression of cyclin E and cyclin B expression exhibited a clear dose-dependent trend, becoming increasingly evident with rising concentrations of 6-Gingerol.

**Table 2:** Induction of cell cycle arrest in ovarian cancer cells and inhibition of cyclin E and cyclin B protein expressions by 6-Gingerol (x ± s, n = 9).

Cell cycle (%)					
Groups	cyclinE	cyclinB	G1	S	G2-M
6-Gingerol 0 mg/L	0.79 ± 0.05	0.69 ± 0.04	51.61 ± 2.28	26.78 ± 1.23	19.64 ± 1.50
6-Gingerol 5.0 mg/L	0.67 ± 0.06*	0.57 ± 0.07*	36.23 ± 3.78*	21.25 ± 3.85*	42.76 ± 3.12*
6-Gingerol 20.0 mg/L	0.57 ± 0.04*	0.49 ± 0.05*	27.56 ± 2.76*	18.67 ± 3.83*	56.68 ± 4.91*
6-Gingerol 40.0 mg/L	0.39 ± 0.05*	0.32 ± 0.04*	15.43 ± 5.32*	11.89 ± 2.50*	72.92 ± 5.37*
F	127.352	96.945	174.08	32.942	318.999
P	0.000	0.000	0.000	0.000	0.000

*p < 0.05.(p, probability value) *F, F-value obtained from one-way ANOVA.

Note: Compare with 6-Gingerol 0 mg/L group.

### 3.3 Induction of apoptosis in ovarian cancer cells by 6-gingerol

**Fig. 2:**
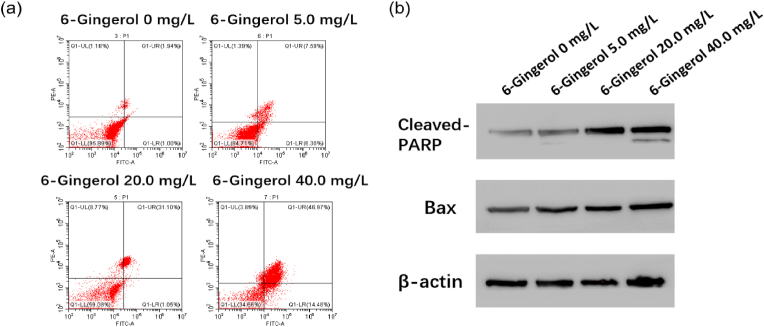
Effects of 6-gingerol on apoptosis-related markers in SKOV3 cells. (a) Representative flow cytometry plots showing apoptosis after treatment with different concentrations of 6-gingerol. (b) Representative Western blot images showing cleaved-PARP and Bax protein expression. β-actin was used as the internal loading control. The corresponding normalized quantitative data are presented in Table 3.

According to Table 3 and Fig. 2, administration of 6-Gingerol led to a significant upregulation of cleaved-PARP and Bax protein levels in SKOV3 cells, along with a pronounced elevation in the apoptotic rate (p < 0.05). Representative Annexin V-FITC/PI flow cytometry plots are shown in Fig. 2a, and the total apoptotic rate was calculated by combining early and late apoptotic cell populations. In addition, the pro-apoptotic effect and the increases in cleaved-PARP and Bax expression displayed a clear concentration-dependent pattern, becoming more prominent with higher doses of 6-Gingerol.

**Table 3:** Induction of apoptosis in ovarian cancer cells and increase of cleaved-PARP and bax protein expressions by 6-Gingerol (x ± s, n = 9).

Groups	Cleaved-PARP	Bax	Cell apoptosis rate(%)
6-Gingerol 0 mg/L	0.04 ± 0.01	0.27 ± 0.02	3.62 ± 0.72
6-Gingerol 5.0 mg/L	0.17 ± 0.03*	0.36 ± 0.04*	8.53 ± 1.81*
6-Gingerol 20.0 mg/L	0.24 ± 0.03*	0.48 ± 0.04*	15.85 ± 3.24*
6-Gingerol 40.0 mg/L	0.41 ± 0.06*	0.68 ± 0.06*	21.36 ± 3.89*
F	127.857	183.823	93.145
P	0.000	0.000	0.000

Note: Compare with 6-Gingerol 0 mg/L group.

*p < 0.05.(p, probability value) *F, F-value obtained from one-way ANOVA.

### 3.4 Effect of 6-gingerol on the PI3K/AKT/mTOR signaling pathway in ovarian cancer cells

**Fig. 3:**
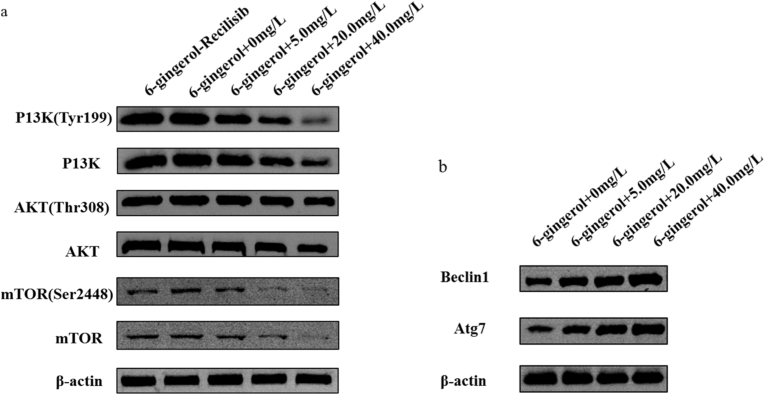
Effects of 6-gingerol on PI3K/AKT/mTOR signaling and autophagy-related proteins in SKOV3 cells. (a) Representative Western blot images showing p-PI3K, total PI3K, p-AKT, total AKT, p-mTOR, and total mTOR expression after treatment with 6-gingerol, with or without Recilisib. Phosphorylated proteins were interpreted relative to their corresponding total protein levels, and β-actin was used as the loading control. (b) Representative Western blot images showing Beclin1 and Atg7 protein expression after treatment with 6-gingerol. β-actin was used as the internal loading control. The corresponding normalized quantitative data are presented in Table 4.

As presented in Fig. 3a, Western blot analysis demonstrated that treatment with 6-Gingerol markedly reduced the ratios of p-PI3K to PI3K, p-AKT to AKT, and p-mTOR to mTOR in SKOV3 cells when compared with the group receiving both 6-Gingerol and Recilisib (p < 0.05). Furthermore, this suppressive effect on the phosphorylation levels of these signaling molecules became increasingly pronounced with escalating doses of 6-Gingerol.

According to Table 4 and Fig. 3b, protein levels of the autophagy-related markers Beclin1 and Atg7 were significantly elevated in SKOV3 cells following 6-Gingerol exposure (p < 0.05). This upregulation also exhibited a clear concentration-dependent pattern, with more substantial increases observed at higher doses of the compound.

**Table 4:** Effect of 6-Gingerol on the expression of Beclin1 and Atg7 protein in ovarian cancer cells (x ± s, n = 9).

Groups	Beclin1	Atg7
6-Gingerol 0 mg/L	0.24 ± 0.05	0.36 ± 0.04
6-Gingerol 5.0 mg/L	0.27 ± 0.05*	0.39 ± 0.03*
6-Gingerol 20.0 mg/L	0.48 ± 0.04*	0.53 ± 0.05*
6-Gingerol 40.0 mg/L	0.69 ± 0.03*	0.61 ± 0.06*
F	283.623	58.101
P	0.000	0.000

Note: Compare with 6-Gingerol 0 mg/L group.

*p < 0.05.(p, probability value) *F, F-value obtained from one-way ANOVA.

### 3.5 Investigating the effect of PI3K/AKT/mTOR pathway activator on the survival of 6-gingerol-Treated ovarian cancer cells

**Fig. 4:**
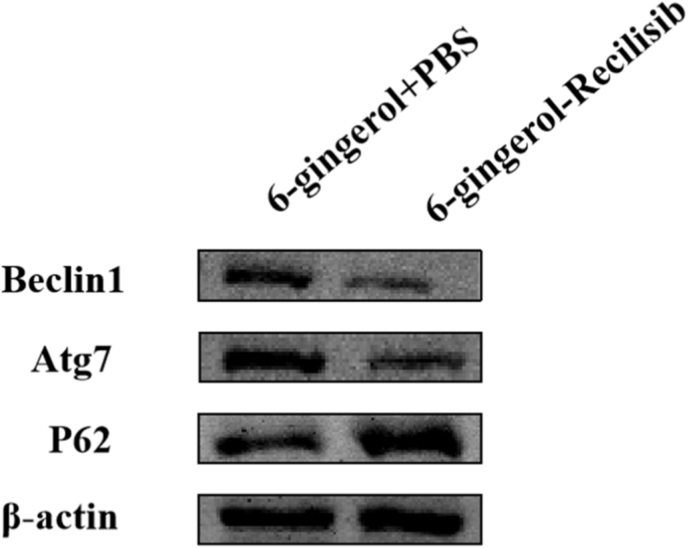
Representative Western blot images showing Beclin1, Atg7, and P62 protein expression in SKOV3 cells treated with 6-gingerol plus PBS or 6-gingerol plus Recilisib. β-actin was used as the internal loading control. The corresponding normalized quantitative data are presented in Table 5.

As shown in Table 5 and Fig. 4, the expression of Beclin1 and Atg7 proteins was significantly reduced, while the expression of P62 was upregulated in SKOV3 cells treated with the 6-Gingerol-Recilisib combination. Additionally, the proportion of apoptotic cells was significantly decreased (*p < 0.05).

**Table 5:** Effects of PI3K/AKT/mTOR pathway activator and 6-gingerol on the proliferation and expression of PI3K pathway proteins in ovarian cancer cells.(x ± s, n = 9).

Groups	Bcelin1	P62	Atg7	Cell apoptosis rate(%)
6-Gingerol + PBS	0.56 ± 0.13	0.36 ± 0.05	0.73 ± 0.05	36.61 ± 2.67
6-Gingerol + Recilisib	0.37 ± 0.05*	0.58 ± 0.078*	0.21 ± 0.04*	15.28 ± 2.62*
F	9.865	8.416	19.117	13.432
P	0.000	0.000	0.000	0.000

Note: Compare with the 6-Gingerol-PBS group.

*p < 0.05.(p, probability value) *F, F-value obtained from one-way ANOVA.

### 3.6 Effect of PI3K/AKT/mTOR activator on apoptosis in 6-gingerol-Treated ovarian cancer cells

**Fig. 5:**
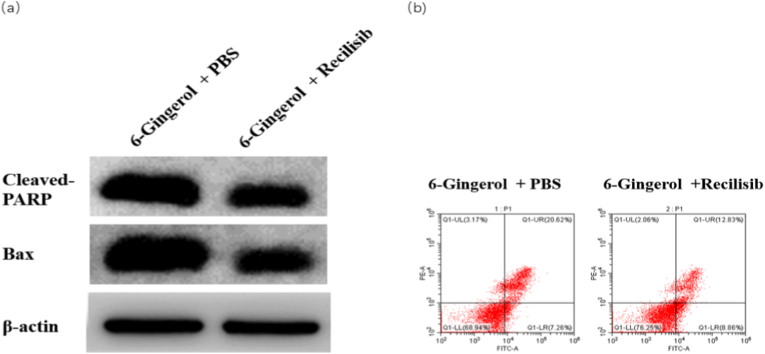
Effects of Recilisib on apoptosis in 6-gingerol-treated SKOV3 cells. (a) Representative Western blot images showing cleaved-PARP and Bax protein expression. β-actin was used as the internal loading control. (b) Representative flow cytometry plots showing apoptosis in SKOV3 cells treated with 6-gingerol plus PBS or 6-gingerol plus Recilisib. The corresponding quantitative data are presented in Table 6.

As shown in Table 6 and Fig. 5, the expression of cleaved-PARP and Bax, as well as the apoptosis rate, were significantly reduced in SKOV3 cells treated with the 6-Gingerol-Recilisib combination (*p < 0.05). Representative Annexin V-FITC/PI flow cytometry plots are shown in Fig. 5b, and quantitative apoptosis data were calculated from the combined early and late apoptotic populations.

**Table 6:** Effect of PI3K/AKT/mTOR activator on apoptosis of 6-Gingerol-treated ovarian cancer cells.(x ± s, n = 9).

Groups	Cleaved-PARP	Bax	Cell apoptosis rate(%)
6-Gingerol + PBS	0.47 ± 0.04	0.73 ± 0.16	17.96 ± 2.47
6-Gingerol + Recilisib	0.19 ± 0.02*	0.27 ± 0.03*	6.17 ± 1.81*
F	18.754	18.435	9.752
P	0.000	0.000	0.000

Note: Compare with a 6-Gingerol-PBS group.

*p < 0.05.(p, probability value) *F, F-value obtained from one-way ANOVA.

## 4 Discussion

6-Gingerol, As the main pharmacologically active component of ginger, 6-gingerol has been widely documented to possess a range of biological properties, such as anticancer, anti-inflammatory, and antioxidant activities [8]. The compound's antitumor effects are thought to stem from its capacity to influence various cellular processes, including apoptosis, regulation of the cell cycle, cytotoxicity, and suppression of angiogenesis [9]. In this study, we showed that 6-gingerol exerts dose-dependent antitumor effects against ovarian cancer cells through the induction of autophagy and cell cycle arrest.

The PI3K/AKT signaling pathway is a central regulatory network involved in cell growth, proliferation, metabolism, survival, and autophagy, and is one of the most frequently dysregulated pathways in cancer. Activation of the PI3K/AKT/mTOR cascade promotes tumor progression and confers resistance to anticancer therapies [10]. Among the three classes of PI3K, Class IA is the most extensively studied and closely associated with oncogenesis. Upon activation, PI3K catalyzes the conversion of PIP_2_ to PIP_3_, leading to AKT recruitment and activation [11]. AKT in turn phosphorylates a variety of downstream substrates, with mTOR serving as the most significant mediator. As a serine/threonine kinase within the PI3K-related kinase family, mTOR acts as a key regulatory hub that suppresses autophagic activity. [12,13]. Suppression of the PI3K/AKT/mTOR signaling cascade is known to trigger autophagy; therefore, this pathway may represent a potential target for therapeutic intervention. [14]. Consistent with this concept, our findings revealed that exposure to 6-gingerol led to a dose-dependent decrease in the phosphorylated levels of PI3K, AKT, and mTOR, aligning with its hypothesized mechanism.

Autophagy represents a core cellular mechanism responsible for preserving homeostasis and genomic integrity through the degradation of damaged organelles and improperly folded proteins. Disruption of autophagic regulation may facilitate tumor development, and targeting this process in malignant cells is increasingly regarded as a viable therapeutic approach. [15]. In our experiments, treatment with 6-gingerol led to dose-dependent changes in the expression of autophagy-related proteins, including increased Beclin1 and Atg7 levels and decreased P62 expression, suggesting a potential association with autophagy activation.Beclin1, which belongs to the autophagy-related (Atg) protein family, is a component of the Class III PI3K complex required for autophagosome formation [16]. Previous reports have suggested that Beclin1 may serve as a potential diagnostic and prognostic indicator in ovarian cancer, with lower expression levels being associated with worse clinical outcomes [17,18]. Similarly, Atg7 is a critical regulator of autophagy initiation and progression, and its loss or dysfunction can impair autophagy and promote tumorigenesis [19]. Our results, which align with earlier reports showing drug-induced Atg7 overexpression promotes autophagy in ovarian cancer [20],suggest that 6-gingerol may influence autophagy-related processes in ovarian cancer cells.

Beyond its influence on autophagy, 6-gingerol also altered cell cycle progression in SKOV3 cells. Dysregulated cell cycle control is a common feature of cancer cells, contributing to their unchecked growth. The G1-to-S and G2-to-M transitions represent critical regulatory nodes that are often targeted for therapeutic purposes. Cyclin E facilitates progression from G1 into S phase by activating CDK2 [21], whereas cyclin B, in complex with CDK1, regulates the G2/M transition [22]. Downregulation of cyclin E disrupts DNA replication initiation and suppresses cell proliferation [23], while reduced cyclin B expression impairs mitotic entry [24]. In our study, 6-gingerol treatment led to significant reductions in cyclin E and cyclin B expression, accompanied by G2-M phase arrest and a concomitant decrease in S and G1 phase cell populations, effects that were more pronounced at higher concentrations, suggesting that 6-gingerol may interfere with cell cycle progression and proliferation-associated cellular processes.

Given the central involvement of the PI3K/AKT/mTOR signaling axis in the progression of ovarian cancer [25] and its status as a recognized target for therapeutic intervention [26], ongoing investigations continue to examine a range of inhibitors targeting this pathway. These include agents that concurrently inhibit PI3K and mTOR, as well as combination regimens integrating such agents with other targeted or conventional treatment modalities [27]. Our findings suggest that 6-gingerol downregulates PI3K/AKT/mTOR signaling, promotes autophagy by upregulating Beclin1 and Atg7, and induces G2-M phase arrest, thereby reducing cell viability, altering cell cycle progression, and promoting apoptosis in ovarian cancer cells. These findings provide preliminary evidence supporting further investigation of 6-gingerol in ovarian cancer research.

## 5 Conclusion

Our investigation demonstrates that 6-gingerol suppresses the PI3K/AKT/mTOR signaling axis in ovarian cancer cells, resulting in G2-M phase arrest, decreased proliferative capacity, and induced apoptosis. These outcomes are partly mediated through enhanced autophagic activity, as evidenced by elevated Beclin1 and Atg7 expression along with reduced P62 levels. The ability of Recilisib—an agonist of the PI3K/AKT/mTOR pathway—to reverse these cellular changes further confirms the critical role of this signaling cascade in mediating the antitumor effects of 6-gingerol.

Collectively,our results provide some mechanistic insights into the biological role of 6-gingerol in ovarian cancer cells. are warranted to evaluate its therapeutic efficacy, optimal dosing regimens, and safety profile, as well as to investigate its potential application in combination strategies targeting the PI3K/AKT/mTOR pathway. However, several limitations exist in our study. First, all in vitro experiments were performed using only the SKOV3 ovarian cancer cell line. Ovarian cancer is a highly heterogeneous disease with diverse histological subtypes, molecular alterations, and biological behaviors; therefore, findings obtained from a single cell line may not be fully representative of ovarian cancer as a whole. Validation in additional ovarian cancer cell lines with different biological backgrounds, as well as comparison with non-cancerous ovarian epithelial cells, would be necessary to determine whether the observed effects of 6-gingerol are broadly applicable and cancer-selective. Due to time and resource constraints, such additional validation could not be completed in the current revision, and this limits the biological and translational interpretation of our findings. Second, autophagy activation was assessed solely by measuring the expression levels of autophagy-related proteins, whereas evaluation of autophagic flux was not performed. Third, although representative Western blot images and available normalized protein-expression data are provided, the absence of complete densitometric bar-graph quantification for all Western blot panels remains a limitation of the present study. Future studies should include full densitometric quantification from original linear-range blot images, together with uncropped blot presentation, to further strengthen the reliability and transparency of protein-expression analysis. Finally, in vivo experiments are lacking to further validate the function of 6-gingerol, which would better simulate its biological effects in animal models. Therefore, further studies using additional ovarian cancer models and animal experiments are required to confirm the underlying mechanisms and therapeutic potential of 6-gingerol.

## Author contributions

Ai-Ying Guo: Conceptualization, Writing – Original Draft.

Yong-Shuai Chen: Writing – Original Draft.

Huan Li: Investigation.

Yun-Yun Ren: Validation.

Xiao-Qing Tan: Formal Analysis.

Xi-Yu Zhang: Visualization.

Jun Xiong: Writing – Review & Editing, Supervision, Funding Acquisition, Project Administration.

## Fund project

This study was supported by the Science and Technology Programme of the Jiangxi Provincial Health and Family Planning Commission (No. 202210036);

The Jiangxi Provincial Natural Science Foundation Senior Project (No. 20242BAB25485), and the Science and Technology Programme of the Jiangxi Provincial Administration of Traditional Chinese Medicine General Project (2024B0268).

## Declaration of competing interest

The authors declare that they have no known competing financial interests or personal relationships that could have appeared to influence the work reported in this paper.

## Data Availability

The datasets used and analyzed during the current study are available from the corresponding author upon reasonable request.
