# Supplementary material for: Effect of 6-gingerol on proliferation and apoptosis of ovarian cancer cells by the PI3K/AKT/mTOR pathway
Source: Biochem Biophys Rep. 2026 Jul 18;47:102717. doi: 10.1016/j.bbrep.2026.102717 (PMC13400410; doi:10.1016/j.bbrep.2026.102717)
Supplement: Multimedia component 1 [file mmc1.docx]

Re: BBREP 102717

Table S1. Antibodies used for Western blot analysis

| \| Target protein \| \| --- \| | Antibody / clone | Manufacturer | Host / isotype | Working dilution for WB |
| --- | --- | --- | --- | --- | --- |
| Cyclin E1 | Cyclin E1 (HE12) Mouse mAb | Cell Signaling Technology | Mouse IgG1 | 1:1000 |
| Cyclin B1 | Cyclin B1 (D5C10) Rabbit mAb | Cell Signaling Technology | Rabbit IgG | 1:1000 |
| Beclin1 | Beclin-1 (D40C5) Rabbit mAb | Cell Signaling Technology | Rabbit IgG | 1:1000 |
| Atg7 | Atg7 (D12B11) Rabbit mAb | Cell Signaling Technology | Rabbit IgG | 1:1000 |
| P62 / SQSTM1 | SQSTM1/p62 (D5E2) Rabbit mAb | Cell Signaling Technology | Rabbit IgG | 1:1000 |
| Bax | Bax (D2E11) Rabbit mAb | Cell Signaling Technology | Rabbit IgG | 1:1000 |
| Cleaved-PARP | PARP-1 / cleaved PARP-1 (194C1439) Mouse mAb | Santa Cruz Biotechnology | Mouse IgG2b | 1:200 |
| p-PI3K | Phospho-PI3 Kinase p85 (Tyr458)/p55 (Tyr199) (E3U1H) Rabbit mAb | Cell Signaling Technology | Rabbit IgG | 1:1000 |
| PI3K | PI3 Kinase p85 (19H8) Rabbit mAb | Cell Signaling Technology | Rabbit IgG | 1:1000 |
| p-AKT | Phospho-Akt (Thr308) (D25E6) Rabbit mAb | Cell Signaling Technology | Rabbit IgG | 1:1000 |
| AKT | Akt (pan) (C67E7) Rabbit mAb | Cell Signaling Technology | Rabbit IgG | 1:1000 |
| p-mTOR | Phospho-mTOR (Ser2448) (D9C2) Rabbit mAb | Cell Signaling Technology | Rabbit IgG | 1:1000 |
| mTOR | mTOR (7C10) Rabbit mAb | Cell Signaling Technology | Rabbit IgG | 1:1000 |
| β-actin | β-Actin (13E5) Rabbit mAb | Cell Signaling Technology | Rabbit IgG | 1:1000 |
| Anti-rabbit secondary antibody | Anti-rabbit IgG, HRP-linked antibody | Cell Signaling Technology | Goat anti-rabbit IgG | 1:2000 |
| Anti-mouse secondary antibody | Anti-mouse IgG, HRP-linked antibody | Cell Signaling Technology | Horse anti-mouse IgG | 1:2000 |
